# Innovative considerations on a phase 2a dose-finding strategy using Bayesian methods and MCP-MOD

**DOI:** 10.1186/1745-6215-16-S2-P204

**Published:** 2015-11-16

**Authors:** Alun Bedding, Julian Zhou

**Affiliations:** 1 Roche Products, Welwyn Garden City, UK; 2 Roche Products, Shanghai, China

## 

Early phase clinical trials in specific patient populations can often have recruitment challenges due to for example a limited disease incidence, concurrent treatment and/or stringent inclusion/exclusion criteria. Owing to this limited availability of patients they often require the use of smaller numbers of patients and more innovative statistical methods. Often single agents, developed for the same disease of interest are not sufficient and may need be used in combination to be effective. Appropriate decsisions may also have to be made for doses, treatment duration and combinations with other compounds.

The plan for the twelve-week phase 2a study in one such population will involve an early futility look at week 2 based on a biomarker, using Bayesian posterior probabilities. This will be followed by using MCP-Mod at the end of study on a clinical response, in order to investigate the dose response.

At week 2, a biomarker is measured to see if there is a clinically meaningful effect at the target for appropriated doses. If not then new doses may need to be added. The posterior probability of the meaningful effect will be used in the decision criteria.

At the end of the study MCP-Mod will be used in order to investiagate the dose response. MCP-Mod involves pre-specifying candidate dose-reponse models followed by statistical testing for dose-response signal whilst controlling the type I error. The best fitting model is then selected, and the target dose estimated, which is then recommended for phase 2b and phase 3 studies. The method has been qualified by the EMA as an efficient method of dose finding.

Simulations have been conducted to investigate the futility and dose finding decisions in phase 2a and understand the operating characteristics, and comparisons made to a traditional dose finding paradigm, which might involve fewer doses using pairwise comparisons to control.

